# Electronic structure of AlFeN films exhibiting crystallographic orientation change from *c*- to *a*-axis with Fe concentrations and annealing effect

**DOI:** 10.1038/s41598-020-58835-5

**Published:** 2020-02-04

**Authors:** Nobuyuki Tatemizo, Saki Imada, Kizuna Okahara, Haruki Nishikawa, Kazuki Tsuruta, Toshiaki Ina, Yoshio Miura, Koji Nishio, Toshiyuki Isshiki

**Affiliations:** 1 Faculty of Electrical Engineering and Electronics, Kyoto Institute of Technology, Kyoto, Kyoto, 606-8585 Japan; 2 Japan Synchrotron Radiation Research Institute, Sayo, Hyogo, 679-5198 Japan; 3 Research Center for Magnetic and Spintronic Materials, National Institute for Materials Science, Tsukuba, Ibaraki, 305-0047 Japan

**Keywords:** Inorganic LEDs, Electronic devices

## Abstract

Wurtzite AlN film is a promising material for deep ultraviolet light-emitting diodes. However, some properties that attribute to its crystal orientation, *i.e*., *c*-axis orientation, are obstacles in realizing high efficiency devices. Constructing devices with non-*c*-axis oriented films is a solution to this problem; however, achieving it with conventional growth techniques is difficult. Recently, we succeeded in growing *a*-axis oriented wurtzite heavily Fe-doped AlN (AlFeN) films *via* sputtering. In this article, we report the electronic structures of AlFeN films investigated using soft X-ray spectroscopies. As-grown films were found to have conduction and valence band structures for a film with *c*-axis in film planes. Simultaneously, it was found that large gap states were formed *via* N-*p* and Fe-*d* hybridization. To remove the gap states, the films were annealed, thereby resulting in a drastic decrease of the gap states while maintaining *a*-axis orientation. We offer heavy Fe-doping and post annealing as a new technique to obtain non-polar AlN films.

## Introduction

Wurtzite III-nitride semiconductors centred on GaN are proven materials for light emitting diodes (LEDs) because they have direct band gaps covering the spectral region of ultraviolet (UV), visible, and infrared lights *via* band gap engineering for the past quarter-century^1–4^. AlN is one of the III-nitrides and it is a promising material for deep UV LEDs^5,6^ by virtue of its wide band gap of 6.1 eV^7^ along with its high thermal conductivity and chemical stability^8^. However, AlN and AlGaN with a high AlN mole ratio confront difficult issues to realize high efficiency devices owing to some electronic properties in the form of thin films, which are traced back to its crystal axis orientations, *i.e. c*-axis orientation.

**Figure 1:**
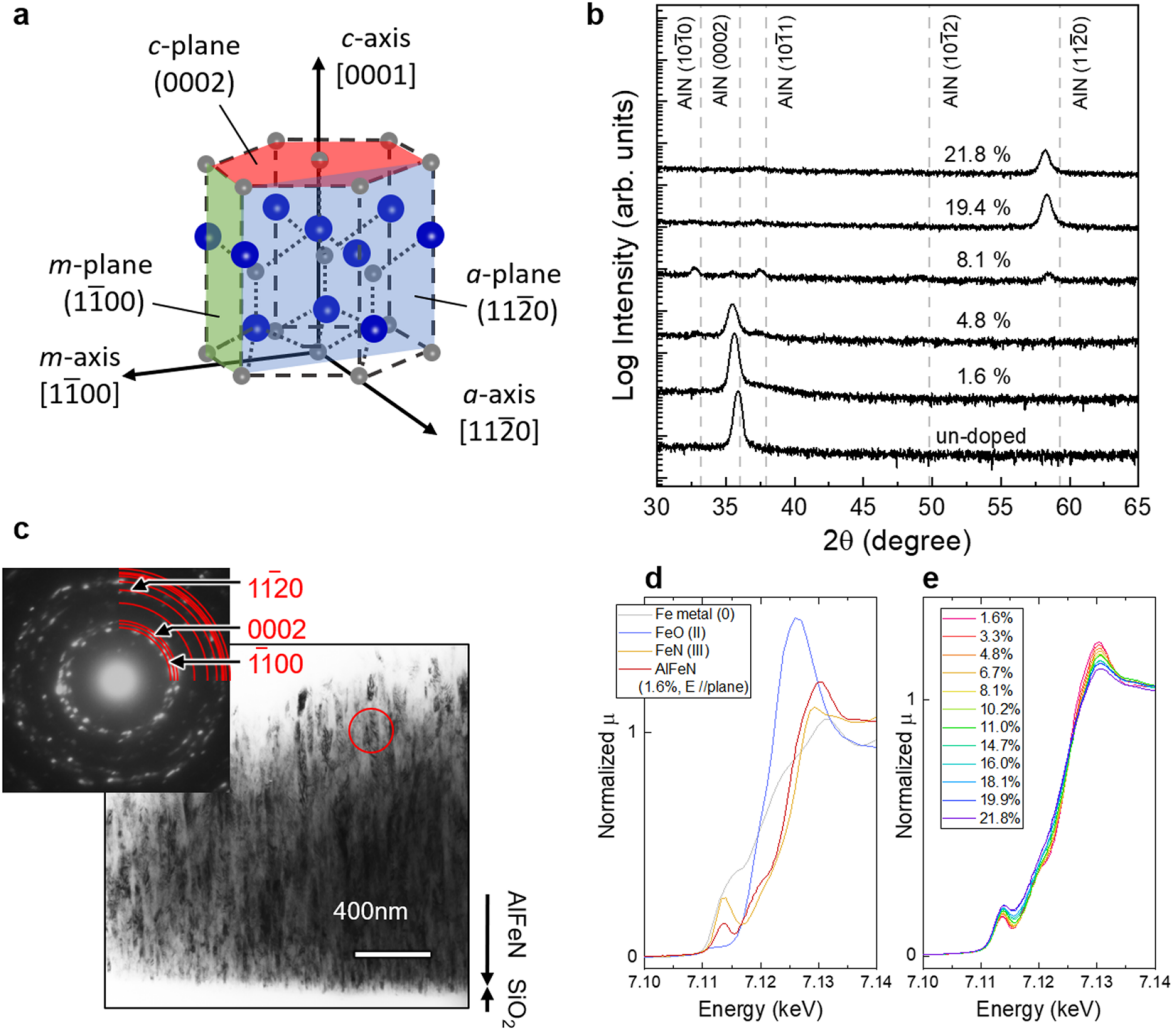
Crystal structure, crystallographic properties of AlFeN films. (**a**) Wurtzite structure with the names of axes and planes. (**b**) Representative XRD profiles of as-grown AlFeN films with an undoped AlN film. (**c**) Cross-sectional TEM images of AlFeN film with 8.1% Fe and corresponding electron diffraction patterns. (**d**) Fe *K*-edge XANES spectra of Fe in AlFeN film (Fe: 1.6%) and standard materials such as Fe metal (0), FeO (II), and FeN (III)^19^. (**e**) Fe concentration dependence of Fe *K*-edge XANES spectra of AlFeN in *E* // plane.

In the *c*-axis direction of a wurtzite structure, electronic polarization can exist owing to a large deference of electronegativities between Al and N, which align along the *c*-axis, as shown in Fig. 1a. Electronic polarization can build internal electric fields in vertical LED structures, which cause low recombination efficiencies due to the quantum confined Stark effect^9,10^. Additionally, the split regimes in the top of valence band (VB) can be another obstacle. Optical dipole transitions between the top of the VB and the bottom of the conduction band (CB) are forbidden for the light with electric fields in the *c*-plane^11^, which result in low extraction efficiency.

Considering the above mechanisms of obstacles for the realization of high efficiency deep UV LEDs, laying *c*-axes in film planes would be a simple solution. Significant efforts have been made to grow films with *m*- and *a*-axes orientations, which are known as non-polar films^12,13^. However, with conventional techniques based on epitaxial growth regimes, there are difficulties in choosing an appropriate substrate^14–17^; moreover, wurtzite films are subjected to the formation *c*-axis orientation.

Very recently, we succeeded in obtaining *a*-axis oriented films via heavily Fe-doping AlN by radio-frequency sputtering^18^. Using this technique, *a*-axis oriented films can be grown with single crystal columnar structures irrespective of the type of substrates, such as SiO_2_ glass and *c*-plane sapphire substrates^18^. In this study, we report the electric structures of AlFeN films analysed by soft X-ray absorption of Al and N *K*-edge and X-ray emission measurements of N *K*-edge. It was found that heavy Fe-doping gives rise to changes in the CB structures that were expected to be in a film with the *c*-axes in the film plane. Simultaneously, however, it was also found that several gap states were formed. *Ab-initio* band structure calculations implied that N-*p* and Fe-*d* hybridizations caused gap states when Fe atoms occupied the Al sites of wurtzite AlN. It is well known that gap states can be the sources of light absorptions and free-carrier killers, which leads to decreased light emitting efficiency. Therefore, we attempted to remove Fe from the AlN lattice *via* thermal annealing. It was shown that the annealing procedure can remove Fe from the lattice and reduce the gap states drastically while keeping the *a*-axis orientation. We offer heavy Fe-doping and post annealing of AlFeN as a promising new route to obtain a seed layer of *a*-axis oriented AlN for high efficiency deep UV LEDs.

## Crystallographic Properties of As-Deposited AlFeN Films

Let us present an overview of the crystallographic properties of as-grown AlFeN films, which will be discussed in this paper. Figure 1b shows the X-ray diffraction (XRD) profiles of representative AlFeN films (Fe: 1.6%, 4.8%, 8.1%, 19.4%, and 21.8%; film thickness: ~2.5 μm) with an undoped AlN film. For the film with low Fe concentration of 1.6%, the profile had a single peak at around 36° similar to that of the undoped AlN, which indicates that the film with 1.6% Fe had a *c*-axis oriented wurtzite structure. At high Fe concentrations of 19.4% and 21.8%, the profiles also had a single peak but at around 58°, a slightly lower angle to wurtzite AlN (11–20). These results indicate that the films had an *a*-axis orientation considering the peak shifts at low angles, which are attributed to the increases in the lattice constants due to Fe-doping. In contrast, in the profiles of the films with 4.8% and 8.1% Fe, multiple peaks appeared. The peaks can be indexed to wurtzite AlN, corresponding to the reflections of (10–10), (0002), (10–11), and (11–20) planes. This indicates that the film has a mixed orientation. Figure 1c shows the cross-sectional transmission electron microscopy (TEM) images of the AlFeN film with 8.1% Fe. The film with 8.1% Fe consists of a columnar structure, similar to the film with 19.4%^18^. As shown in the inset of Fig. 1c, electron diffraction patterns in the circled area in Fig. 1c showed multiple spots, such as 1–100, 0002, and 11–20, implying that the columnar grains have different orientations such as *m*, *c*, and *a*, unlike the film with 19.4% Fe, where every columnar had an *a*-axis orientation^18^. This result is consistent with the result of XRD measurements.

Fe *K*-edge X-ray absorption near edge structure (XANES) measurements were conducted to reveal the local crystallographic and electronic structures of Fe in AlFeN films. Figure 1d depicts the Fe *K*-edge XANES spectra of Fe in AlFeN film (Fe: 1.6%) and standard materials such as Fe metal (0), FeO (II), and FeN (III)^19^. The main edge energy of Fe in AlFeN appears close to that of FeN (III). A pre-edge peak was clearly observed in the AlFeN film spectrum, similar to FeN where an Fe atom is surrounded by four nitrogen atoms with non-centrosymmetric condition of the zincblende structure^20^. These results imply that most Fe atoms in the film with 1.6% Fe have an oxidation state close to 3+ and occupy a site with non-centrosymmetric conditions, suggesting that Fe atoms in the AlFeN film occupy the Al sites of a wurtzite structure. Figure 1e shows the spectra of AlFeN films with various Fe concentrations. The main absorption edge energies and the existence of pre-edge peaks did not show a dependency on the Fe concentration. These results suggest that the Fe atoms in the AlFeN films occupy the Al sites of a wurtzite structure, irrespective of the orientations of the films.

## Electronic Structure of As-Deposited AlFeN Films

**Figure 2:**
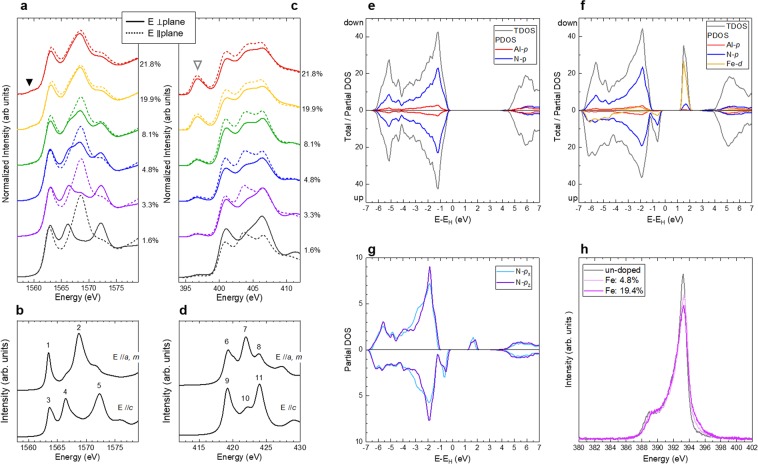
Experimental XANES and XES spectra, theoretical XANES and band structures. (**a**) Al *K*-edge XANES spectra of AlFeN films measured in *E* ⊥ plane and *E* // plane modes. (**b**) Theoretical Al *K*-edge XANES spectra of AlN in *E* //*a*- and *m*-axes and *E* //*c*-axis. (**c**) N *K*-edge XANES spectra of AlFeN films measured in *E* ⊥ plane and *E* // plane modes. (**d**) Theoretical N *K*-edge XANES spectra of AlN in *E* //*a*- and *m*-axes and *E* //*c*-axis. (**e**) Theoretical TDOS and PDOS of Al-*p* and N-*p* for AlN. (**f**) Theoretical TDOS and PDOS of Al-*p*, N-*p*, and Fe-*d* for Al_33_Fe_3_N_36_. (**g**) Theoretical PDOS of N-*p*_x_ and N-*p*_z_ for Al_33_Fe_3_N_36_. The zeros in every energy axis (*E*-*E*_H_) of DOS are the highest occupied states by electrons at ground state. (**h)** Experimental XES spectra for AlFeN films with 4.8% Fe and 19.4% Fe with undoped AlN film.

Al and N *K*-edge XANES spectra reflect the electron-unoccupied partial density of states (PDOSs) of Al-*p* and N-*p*, respectively, which are the main components of the bottom of the CB of AlN. When the incident X-ray is linearly polarized, XANES spectra reflect the PDOSs with the same direction as the electric field *E* of the incident X-ray. Figure 2a,c show the Al and N *K*-edge XANES spectra of the as-grown films, respectively, obtained by the total electron yield mode at UVSOR in Okazaki, Japan. The solid and broken lines were obtained when the electric field vectors *E* of the incident X-rays were perpendicular and parallel, respectively, to the film plane. To analyse the experimental spectra, we also performed theoretical calculations on the XANES spectra for a wurtzite AlN using the FDMNES code^21,22^. Figure 2b shows the simulated Al *K*-edge XANES spectra for *E* //*a, m* and *E* //*c*. In the simulated spectra, clear differences between *E* //*a, m* (the spectra for *E* //*a* and *E* //*m* are degenerated) and *E* //*c* appeared; peak 2 is characteristic for *E* //*a, m* and peak 4 and peak 5 are characteristic for *E* //*c*, while peak 1 and peak 3 commonly appear for both *E* //*a*, *m* and *E* //*c*. These features are significantly consistent with those previously reported, both theoretically^23^ and experimentally^24^. The experimental spectra of AlFeN with 1.6% Fe in Fig. 2a are significantly consistent with the theoretical spectra; the spectra for *E* ⊥ plane and *E* // plane are coincident with the theoretical spectrum for *E* //*c* and *E* //*a*, *m*, respectively. This result is consistent with the XRD result, indicating that the film with 1.6% Fe had *c*-axis orientation.

For the film with 3.3% Fe, slight sign of peak 2 appeared in the spectrum for the *E* ⊥ plane, which indicated that a small *a*- and/or *m*-axes oriented region grew in the film. For the *E* ⊥ plane, peak 5 became slightly small, which indicated that the volume of *c*-axis oriented region became small. Considering that no obvious peak, except the (0002) peak, appeared in the XRD profile of the film with 3.3% Fe, the volumes of the non-*c*-axis oriented regions were smaller than the detection limits of XRD measurements. By increasing the Fe concentrations up to 8.1%, these tendencies were enhanced, which suggests that the volume of the *c*-axis oriented regions became small with Fe concentrations. These results are consistent with the XRD results. At 19.9% and 21.8% Fe, the spectra for *E* ⊥ plane and *E* // plane resembled each other. Because the *a*-axis oriented AlFeN films have random orientation in plane^18^, all directions between the *c*-axis and *m*-axis can be parallel to the vector *E* for the *E* // plane mode, while *a*-axis is parallel to the vector *E* for the *E* ⊥ plane mode. In this case, the strongest peak 2, which is characteristic for *a*- and *m*-axes, and peak 1 and peak 3, which are a common characteristic in all directions, can appear in both modes (*E* // plane and *E* ⊥ plane). It should be noted that the Fe substitution for Al might affect the structure of CB at such high Fe concentrations. This will be discussed after an overview of the N *K*-edge XANES spectra.

Figure 2c shows the experimental N *K*-edge XANES spectra of AlFeN films. The theoretical spectra of N *K*-edge XANES for AlN are depicted in Fig. 2d. The *E* direction dependence of AlFeN with 1.6% Fe can be interpreted as a result of *c*-axis orientation. The Fe concentration dependence of the N *K*-edge spectra can also reflect the change in the orientation, similar to the Al *K*-edge XANES analyses. Noteworthy findings in N *K*-edge spectra are the formation of the so-called pre-edge peaks at around 396 eV, indicated by the open triangle in Fig. 2c. Because the N atoms are thought to be bounded to Fe atoms directly, changes in N-*p* PDOS might occur through hybridization with Fe-*d* orbitals under the situation in non-centrosymmetric conditions.

To test the assumption described above, PDOSs of Al-*p*, N-*p*, and Fe-*d* for Al_36−x_Fe_x_N_36_ with total density of states (TDOS) for x = 0 (un-doped) and 3 (8.3% Fe) were calculated using the Vienna *ab-initio* simulation package (VASP)^25,26^ code and are depicted in Fig. 2e,f. The electron unoccupied states consisted of mainly Fe-*d*, and N-*p* was formed in the middle of the energy gap. These results support the assumption of the origin of the pre-edge peaks in the N *K*-edge XANES spectra. As shown in Fig. 2g, the shapes and numbers of N-*p*_x_ and N-*p*_z_ are similar. This is considered the reason for the similarity in the relative shapes and strengths of the pre-edge peaks and the main peaks of the *E* // plane and *E* ⊥ plane spectra. As shown in Fig. 2f, Al-*p* components also have gap states, which, however, are very small. This can be a reason why the pre-edge peaks in the Al *K*-edge XANES spectra are visible only at high Fe concentrations of 19.9% and 21.8%.

In the electron-occupied DOS in AlFeN, the gap states lay directly above the top of VB, which consists of mainly N-*p* and Fe-*d*. To analyse the electron-occupied structure, X-ray emission spectroscopy (XES) for N *K*-edge was conducted and plotted in Fig. 2h. In the spectra of AlFeN films with 4.8% and 19.4%, small but clear signals between 394 eV and 396 eV were observed just above the main structures between 388 eV and 394 eV. This result implies that the electron occupied states were formed as suggested by the theoretical calculation. Owing to the weak anisotropy of the N-*p* PDOS structure (see Fig. 2g), in conjunction with the low resolution of the XES measurements compared to that of the XANES measurements, no obvious difference was observed in the shapes of the main structures between the AlFeN films of 4.8% and 19.4% Fe, which have *c*- and *a*-axis orientations, respectively.

## Annealed AlFeN Films

**Figure 3:**
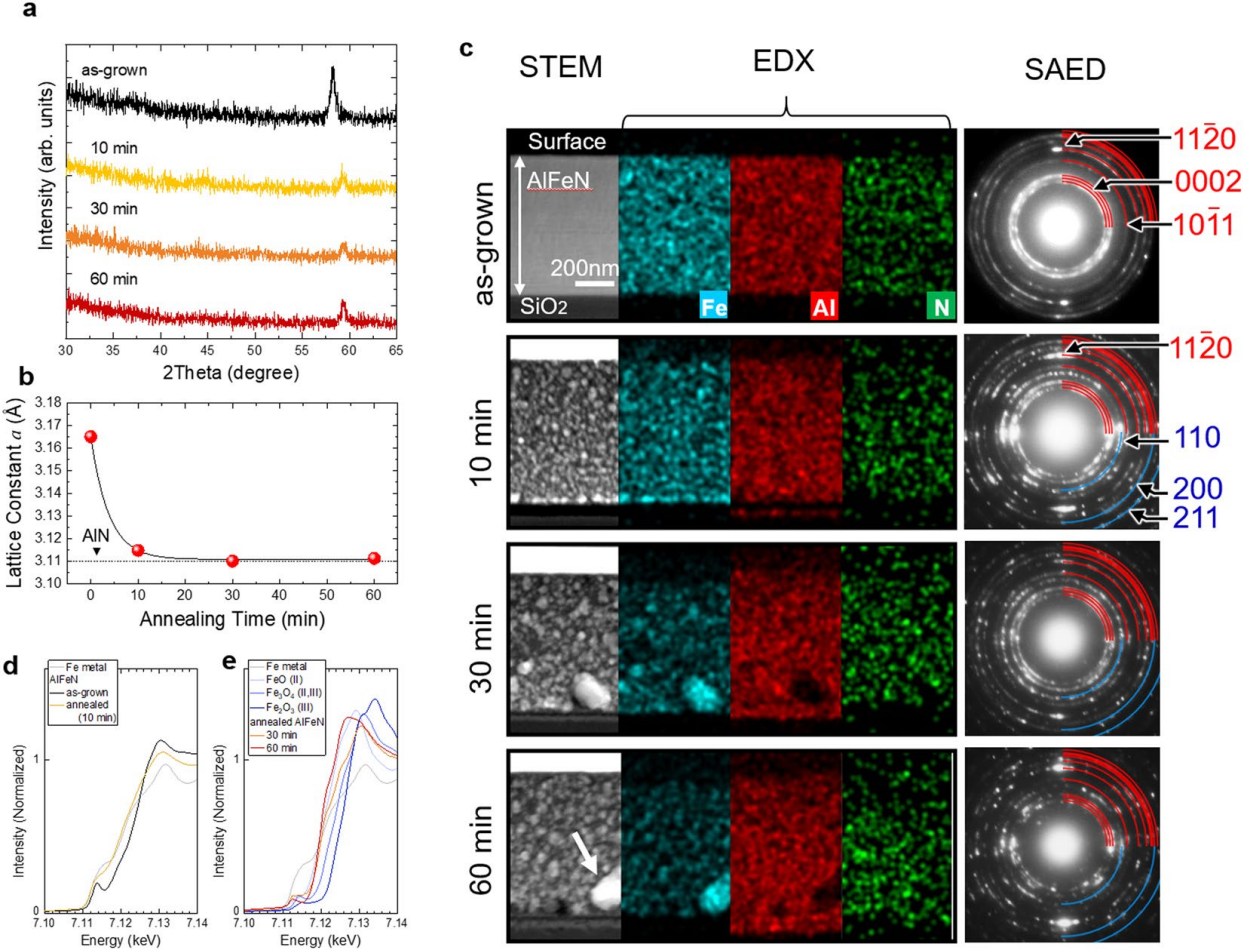
Crystallographic properties of annealed AlFeN films. (**a**) XRD profiles of as-grown and annealed AlFeN films. (**b**) Annealing time dependence of lattice constant *a* estimated from the XRD profiles. (**c**) ZC STEM images, maps of Fe, Al, and N, with selected area electron diffraction (SAED) patterns corresponding to the STEM images of as-grown and annealed AlFeN films. (**d**) Fe *K*-edge XANES spectra of as-grown and annealed AlFeN films with Fe metal measured using the conversion electron yield method. (**e**) Fe *K*-edge XANES spectra of annealed AlFeN films with various Fe oxides.

As suggested above, as-grown AlFeN films have CB and VB structures with *c*-axis in film planes whereas large gap states were formed *via* N-*p* and Fe-*d* hybridization. Thus, Fe atoms in the Al sites of an AlN lattice are considered to be one of the origins of gap-states, which might cause low efficiency in LEDs when the LED structure is constructed on an AlFeN seed layer. To decrease the gap states, thermal annealing was conducted to remove the Fe atoms in an AlN lattice of the *a*-axis oriented AlFeN samples. The Fe concentration and film thickness were around 19.6% and 0.6 μm, respectively. The annealing temperature was 1200 °C. During the annealing process, the film surface was covered with an SiO_2_ glass under N_2_ gas flow. Figure 3a shows the annealing time dependence of the XRD profiles. All films were shown to have single peaks between 58° and 59° of 2*θ*, suggesting that the films keep an *a*-axis oriented wurtzite structure. The peak positions of the annealed films slightly shifted toward a higher degree than those of as-grown films. The lattice constant *a* was estimated from the peaks and plotted in Fig. 3b. The lattice constant *a* decreased drastically after annealing for 10 min and then became close to that of AlN for longer annealing. This is due to the elimination of Fe from the wurtzite lattice. This tendency is opposite to that of doping, which increases the lattice constants. Fe segregation was confirmed by cross-sectional scanning TEM (STEM) and energy dispersive X-ray (EDX) mapping analyses. Figure 3c shows the atomic number, Z, contrast (ZC) STEM images, maps of Fe, Al, and N, and SAED patterns of as-grown and annealed samples. A ZC image can reflect the distribution of atoms: a bright point originates from heavy atoms and a dark point originates from light atoms. The ZC image of an as-grown sample shows uniformity in contrast, while bright regions appeared in those of annealed samples. This result implies that Fe, which is the heaviest atom among Fe, Al, and N, segregated in the film. In the EDX map of Fe, agglomerations of bright signals were observed especially near the substrates of the annealed film, while only very weak signals were detected in the regions of maps of Al and N. It was confirmed by EDX analyses that the composition of the large bright region, indicated by the white arrow, was approximately 100% Fe. The SAED patterns support these results; additional spots were detected in the annealed samples corresponding to 110, 200, and 211 from α-Fe. Here, a noteworthy discovery is that the 11–20 of wurtzite structure spots along the growth direction remained strong in the SAED patterns of the annealed samples, thus indicating that the films maintain *a*-axis orientation in the wurtzite structure. This is consistent with the results of the XRD analyses. As for the segregated α- Fe, there were no corresponding peaks in the XRD profiles of the annealed samples. The reason for this may be attributed to the fact that each region of the α- Fe had a smaller volume than the detection limit of the XRD measurements.

To investigate the states of Fe near the surface, we conducted Fe *K*-edge XANES measurements in the conversion electron yield mode, which is a surface sensitive method. Figure 3d shows the spectra of as-grown and annealed (10 min) AlFeN films and Fe metal. The spectrum of AlFeN annealed for 10 min has a similar shape as that of the Fe metal. After a longer annealing time, the spectra showed a sharp rise in the main edges, such as FeO (II), as depicted in Fig. 3e. These results imply that some Fe atoms escaped the lattice sites and were oxidized near the surface during the long annealing time. SiO_2_ glass is considered to be the source of oxygen, which covered the sample surface during annealing and/or residual oxygen in the annealing atmosphere.

**Figure 4:**
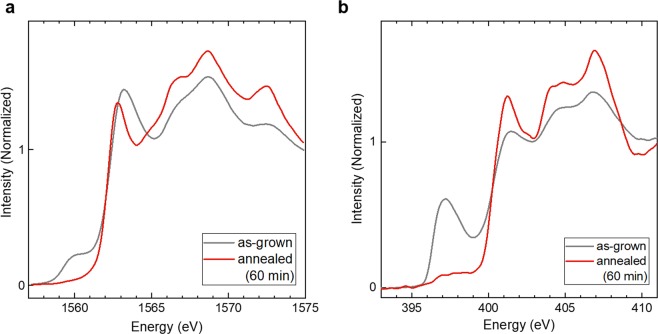
XANES spectra of annealed films. (**a**) Al *K*-edge XANES spectra of as-grown and annealed films. (**b**) N *K*-edge XANES spectra of as-grown and annealed films.

Figure 4a,b show the Al and N *K*-edge XANES spectra, respectively, measured for *E* // plane in the fluorescent mode, which is bulk sensitive. In the Al *K*-edge XANES spectrum of the annealed film, the following remarkable changes were observed: (i) the main peaks of the annealed film kept a structure observed in those of the as-grown film, while every peak became sharp and (ii) the intensities of the pre-edge peaks drastically decreased. Determining (i) implies that the annealed film has a CB structure with *c*-axis in the film plane. The sharp peaks might be due to the decrease in hybridization with Fe orbitals. Determining (ii) implies that the gap states can be annihilated by removing Fe from the lattice by annealing. Similar changes corresponding to (i) and (ii) were observed in the N *K*-edge XANES spectrum of the annealed film. In conjunction with the XRD results, these XANES results suggest that the annealed films keep their *c*-axis parallel to the film planes.

## Conclusion

The electronic structures in the vicinity of CB and VB of AlFeN films were investigated by Al and N *K*-edge XANES. It was found that as-grown films have electronic structures, which are expected in *a*-axis oriented films, while gap-states were formed in the gap. To remove the gap-states that cause low efficiency, the films were annealed, thereby resulting in a drastic decrease in the gap-states while maintaining an *a*-axis orientation. It was shown that the inclusions of Fe metals remained in the annealed films. Such inclusions in the seed layer could be sources of light absorptions, free-carrier killers, and electrical leakage, which may lead to decreased light emitting efficiency in an LED. It was also implied that Fe atoms remained in the surface region of the films as Fe-oxides, which should be clean when the films are used as a seed for non-polar LEDs. Thus, it is necessary to optimize the annealing procedures that can remove Fe inclusion and maintain the surface free from oxides. As already reported in our paper^18^, the *a*-axis oriented AlFeN films have random orientation in the plane. The annealed films also exhibit random orientation in the plane, as suggested by the Al and N *K*-edge XANES analyses. To use the films as seed layers for non-polar LEDs, we propose two directions to develop our findings: obtaining single crystal films as seed layer for conventional LEDs and obtaining films comprising homogeneous pillars without a tilt in the *a*-plane for nanocolumn-LEDs^27,28^. Once these are achieved, heavy Fe-doping and post annealing can be used as a new technique to obtain a seed layer to realize non-polar AlN devices.

## Methods

### Synthesis of AlFeN films

AlFeN films were deposited on SiO_2_ glass substrates directly by radio frequency sputtering (ULVAC KIKO, Inc., RFS-200S) from an AlN target (Furuuchi Chemical, 99%) with Fe metal chips (Furuuchi Chemical, 99.9%) on it. The deposition temperature was 300 °C and a flow of Ar/N_2_ mixture (2: 1) at a pressure of 0.65 Pa was used (both are of 6 N purity from Iwatani Gas). The thicknesses of the prepared films were approximately 2.5 μm for the electronic structure analyses of as-grown films and approximately 0.6 μm for the annealing experiments. Fe concentrations were determined using energy dispersive X-ray fluorescence analysis (HITACHI High-Technologies, SEA5120). The Fe concentration is expressed in atomic percent with respect to the total cation content.

### Annealing of AlFeN films

An infrared image furnace (ULVAC RIKO, Inc., MIRA-5000) was used to anneal the samples. The annealing temperature was 1200 °C and a flow of N_2_ gas at atmospheric pressure was used (4 N purity, Iwatani Gas).

### XRD measurements

X-ray diffraction (XRD) measurements were conducted using Cu *Kα* in the out-of-plane mode (PANalytical, X’Pert MRD) at room temperature (RT).

### TEM specimen preparation and imaging

Samples for transmission electron microscopy (TEM) were prepared by mechanical polishing, followed by ion-thinning methods. Cross-sectional TEM images were obtained using a JEOL JEM-2010 system at 200 kV.

### Fe *K*-edge XANES measurements

Fe *K*-edge X-ray absorption near edge structure (XANES) measurements in the fluorescence mode for as-grown AlFeN films were conducted at the BL12C of the KEK photon factory (KEK-PF). For annealed AlFeN films, the measurements were performed in conversion electron detection mode at the BL01B1 of SPring-8. The electric field vectors *E* of the incident X-rays were parallel to the film plane. All measurements were performed at RT.

### Al and N *K*-edge XANES spectroscopy

N and Al *K*-edge XANES measurements of the as-grown films were performed at the BL4B and BL2A of UVSOR, respectively, in the total electron yield mode. The electric field vectors *E* of the incident X-rays were parallel and perpendicular to the film plane, *E* // plane, and *E* ⊥ plane, respectively. For annealed AlFeN films, the measurements were performed at the BL27SU of SPring-8 in the fluorescence-detection mode. This mode was chosen because the electric resistivities of the annealed films were very high and thus, the total electron yield mode could not be applied. The electric field vectors *E* of the incident X-rays were parallel to the film plane. All measurements were performed in vacuum ~10^−7^ Pa at RT.

### N *K*-edge XES spectroscopy

N *K*-edge X-ray emission spectroscopy (XES) of the as-grown films was performed at the BL27SU of SPring-8. The excitation energy was 430 eV, which is above the absorption threshold of N *K*-edge. The electric field vectors *E* of the incident X-rays were parallel to the film plane. The XES spectra were recorded in a 90° scattering geometry. All measurements were performed in vacuum ~10^−7^ Pa at RT.

### Theoretical XANES spectra calculation

The Al and N *K*-edge XANES spectra of wurtzite AlN were calculated using the *ab-initio* FDMNES^21,22^ code with full potential. The internal parameter of the wurtzite structure was 0.382. The lattice constants of the undoped AlN films obtained from the XRD measurements were applied to the model (*a* = 3.08675 Å and *c* = 5.03276 Å). The cluster radius for the calculation was 7 Å (approximately 144 atoms were included).

### Theoretical band structure calculation

TDOS and PDOS of Al_33_Fe_3_N_36_ were calculated using the first-principle density functional calculations using the VASP^25,26^. For the exchange and correlation energies, we adopted the spin-polarized generalized-gradient approximation with Perdew-Becke-Ernzerhof parameterization^29^. In the calculations, we used bulk 3*a* × 3*a* × 2*c* periodic supercells including 33 Al, 3 Fe, and 36 N atoms in the wurtzite structure. In this study, the lattice constants of the AlFeN films obtained from the XRD measurements were applied in the periodic supercells (*a* = 3.14863 Å and *c* = 5.07895 Å). The Hubbard correction was applied to Fe with U^30^ parameter equal to 2 eV in Fig. 2f. (Refer to the supplemental for why U = 2 was selected in Fig. 2f.) The atomic positions were relaxed. The Brillouin zone integration was performed on a uniform 6 × 6 × 5 *k*-point mesh. The nuclei and core electrons were described by the projector augmented plane-wave potential^31^ and the wave functions were expanded in a plane-waves basis set with a cut-off energy of 408 eV.

### STEM/EDX

Cross-sectional STEM and EDX images were obtained using STEM (Hitachi High-Technologies, HD-2700) operating under 200 kV, equipped with SDD (Bruker, QUANTAX200STEM). Cross-sectional samples were prepared using the focused ion beam system (Hitachi High-Technologies, FB-2200) with a Ga ion beam.

## Supplementary information

Figure S1.
