# Supplementary material for: Electronic structure of AlFeN films exhibiting crystallographic orientation change from c- to a-axis with Fe concentrations and annealing effect
Source: Sci Rep. 2020 Feb 4;10:1819. doi: 10.1038/s41598-020-58835-5 (PMC7000734; doi:10.1038/s41598-020-58835-5)
Supplement: Supplementary file 1 — Figure S1. [file 41598_2020_58835_MOESM1_ESM.pdf]

Electronic structure of AlFeN films exhibiting crystallographic orientation change from *c*- to *a*-axis with Fe concentrations and annealing effect

Nobuyuki Tatemizo[1*], Saki Imada[1*], Kizuna Okahara[1], Haruki Nishikawa[1], Kazuki Tsuruta[2], Toshiaki Ina[2], Yoshio Miura[3], Koji Nishio[1], and Toshiyuki Isshiki[1]

[1]Faculty of Electrical Engineering and Electronics, Kyoto Institute of Technology, Kyoto, Kyoto 606-8585, Japan

[2] Japan Synchrotron Radiation Research Institute, Sayo, Hyogo 679-5198, Japan

[3] Research Center for Magnetic and Spintronic Materials, National Institute for Materials Science, Tsukuba, Ibaraki 305-0047, Japan

*e-mail: n-tatemizo@kit.ac.jp, saki_imada@kit.ac.jp

Supplemental:

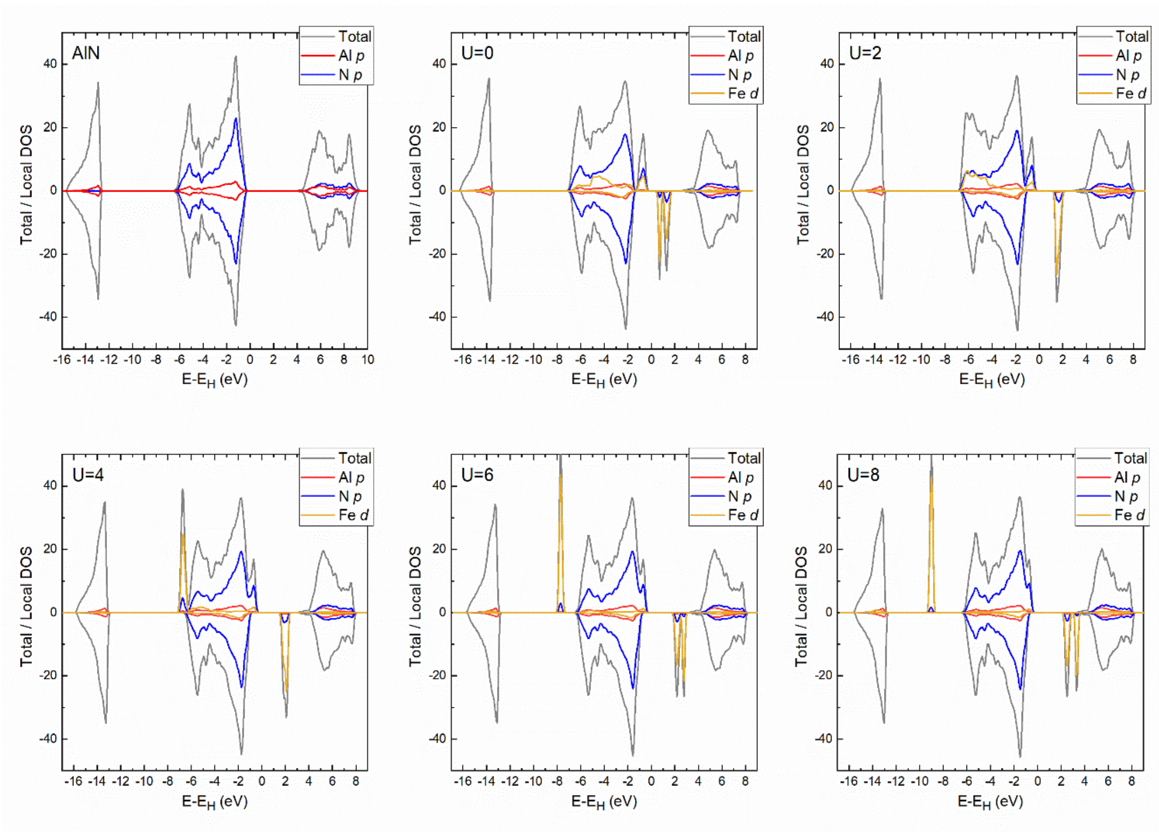

Fig. S1

Figures S1a-S1f show the calculated band structures using the VASP code for $Al_{33}Fe_3N_{36}$ for various Hubbard corrections (U) for Fe from U = 0–8. The zeros in each energy axis ($E-E_H$) are the highest occupied states by the electrons at ground state. In the N-*p* PDOS for U = 4, 6, and 8, a peak appeared just under the main component of VB between 0 and -6 eV. Because there was no corresponding peak in the XES, we chose U = 2 for Fig. 2f. To determine the Hubbard corrections (U) for Fe as well as Al and N, other experiments such as Fe *L*-edge XANES and XES include resonant XES and Al *K*-edge XES.
